# Standardized Video Interview Scores Correlate Poorly with Faculty and Patient Ratings

**DOI:** 10.5811/westjem.2019.11.44054

**Published:** 2019-12-19

**Authors:** Matthew M. Hall, Jason J. Lewis, Joshua W. Joseph, Andrew R. Ketterer, Carlo L. Rosen, Nicole M. Dubosh

**Affiliations:** Beth Israel Deaconess Medical Center, Harvard Medical School, Boston, Massachusetts

## Abstract

The Standardized Video Interview (SVI) was developed by the Association of American Medical Colleges to assess professionalism, communication, and interpersonal skills of residency applicants. How SVI scores compare with other measures of these competencies is unknown. The goal of this study was to determine whether there is a correlation between the SVI score and both faculty and patient ratings of these competencies in emergency medicine (EM) applicants. This was a retrospective analysis of a prospectively collected dataset of medical students. Students enrolled in the fourth-year EM clerkship at our institution and who applied to the EM residency Match were included. We collected faculty ratings of the students’ professionalism and patient care/communication abilities as well as patient ratings using the Communication Assessment Tool (CAT) from the clerkship evaluation forms. Following completion of the clerkship, students applying to EM were asked to voluntarily provide their SVI score to the study authors for research purposes. We compared SVI scores with the students’ faculty and patient scores using Spearman’s rank correlation. Of the 43 students from the EM clerkship who applied in EM during the 2017–2018 and 2018–2019 application cycles, 36 provided their SVI scores. All 36 had faculty evaluations and 32 had CAT scores available. We found that SVI scores did not correlate with faculty ratings of professionalism (rho = 0.09, p = 0.13), faculty assessment of patient care/communication (rho = 0.12, p = 0.04), or CAT scores (rho = 0.11, p = 0.06). Further studies are needed to validate the SVI and determine whether it is indeed a predictor of these competencies in residency.

## BACKGROUND

In 2017, the Association of American Medical Colleges (AAMC) developed the Standardized Video Interview (SVI) score as an additional way to assess the professionalism, communication, and interpersonal skills of residency applicants.^1^ The SVI is composed of six questions answered via a video-recorded, computerized interface and centered on two core competencies of the Accreditation Council for Graduate Medical Education (ACGME): knowledge of professional behavior and interpersonal and communication skills.^2^ Responses are scored by third-party reviewers using a 1–5 point system with a composite score of 6–30 (Appendix A).^3^ This score was provided in the 2017–2018 and 2018–2019 Electronic Residency Application Service (ERAS) application packets for emergency medicine (EM) residencies.

The AAMC and several leading EM organizations have sought to assess the validity the SVI. The AAMC found that SVI scores did not correlate with United States Medical Licensing Examination scores and speculated that they would add an additional element to the application.^4^ The decision was made to proceed with a pilot administration during the 2017–2018 application period.^5^ While the SVI may add additional information to the residency application, it is unclear how it correlates with other measures of professionalism and communication. Previous work has shown that the SVI does not correlate with faculty gestalt of communication and professionalism.^6^ We sought to investigate whether correlations exists between the SVI and two other measures of these competencies in EM applicants: faculty end-of-shift ratings of patient care/communication and professionalism, and patient ratings of communication skills.

## OBJECTIVES

The goal of this study was to determine whether a correlation exists between the SVI and faculty and patient ratings of these competencies in EM applicants. This was a retrospective analysis of a prospectively collected dataset including fourth-year medical students who enrolled in the EM clerkship at our institution and applied to EM residencies in 2017–2018 and 2018–2019. We collected self-reported SVI scores, end-of-shift faculty evaluations on professionalism and patient care/communication, and scores on the Communication Assessment Tool (CAT), a questionnaire assessing communication skills from the patient perspective that has validity evidence.^7^–^9^ We compared scores on all three tools using Spearman’s rho. Statistical analyses were performed with Python 3.6 (Python Software Foundation, Fredericksburg, VA).^10^–^11^ A p-value of <0.05 with a Bonferroni correction for multiple comparisons was considered statistically significant. This study was determined to be exempt by our institutional review board.

## RESULTS

Forty-three students from our EM clerkship applied to EM during the study period. The response rate of SVI scores was 86.7% (36/43). Fifty-eight faculty members completed evaluations. Faculty ratings were available for 36 students, and CAT scores were available for 32 students. Median scores are shown in Table 1. None of the three tools had a normal distribution (p<0.01). SVI scores did not correlate with CAT scores (rho = 0.11, p = 0.06), nor with faculty evaluation of professionalism (rho = 0.09, p=0.13) or patient care/communication (rho = 0.12, p = 0.04). Faculty professionalism and patient care/communication scores were highly correlated (rho = 0.86, p<0.05).

**Population Health Research Capsule**

What do we already know about this issue?

*The SVI does not correlate with faculty gestalt of communication and professionalism*.

What was the research question?

Is there a correlation between the SVI and faculty and patient ratings of professionalism and communication skills?

What was the major finding of the study?

*We found no correlation between the SVI and ratings of professionalism and communication skills*.

How does this improve population health?

*This project suggests further research into the SVI is warranted before full implementation of this assessment tool*.

## IMPACT

We found no significant correlation between students’ SVI scores and faculty ratings of professionalism and patient care/communication skills or CAT scores. To the best of our knowledge, this is the first study comparing SVI scores with existing measures of communication and professionalism in the clinical setting.

Assessing communication and professionalism skills is essential in medical training, and the ACGME has identified both as core competencies.^12^ A recent review demonstrates that EM program directors value strong interpersonal and humanistic qualities in applicants.^13^ While it is important to understand applicants’ professionalism and communication abilities, there is currently no “gold standard” assessment method. The ACGME suggests multi-source feedback and multiple evaluators for assessing trainees’ competencies.^14^ While validated tools are still needed, the use of multi-source assessment including patient feedback in the clinical setting has been shown to be successful.^15^,^16^ The SVI scenarios are neither real-time clinical scenarios nor interactions with patients, and it is unclear whether an artificial testing environment is the ideal method of evaluating these competencies. The lack of correlation between the SVI and real-time evaluation of patient interactions raises questions about the SVI’s validity. While the SVI is no longer being considered for use in EM, understanding the concerns surrounding its validity is essential if it is to be reconsidered in the future or used in other specialties.

## LIMITATIONS

Our study has several limitations. The SVI scores are self-reported; thus, it is possible students did not provide the correct score. We used this methodology given proprietary restrictions regarding the use of ERAS data. Second, as a single-center study with a small sample size, generalizability is limited. Larger studies are needed to confirm these findings. While students worked 14 clinical shifts during their clerkship, the median number of faculty evaluations completed for each student was nine. This faculty response rate may have introduced bias to these scores. While faculty at our institution are offered individualized training by the clerkship directors on completing evaluations, it is possible that not all faculty participated in a training session and inter-rater reliability may be limited.

Additionally, the faculty evaluation tool groups patient care and communication together (Appendix B), and it is possible some faculty may have weighed this domain more heavily on the patient care aspect and not communication. Four of the students’ CAT scores were lost and not included in the analysis; however, there were no demographic differences between these students and the analyzed population, and thus we do not expect this to have skewed the results. Neither the CAT nor our faculty evaluation system has been validated in terms of predicting success in residency; therefore. we cannot draw conclusions about the SVI’s utility at assessing residency success based on our data. However, there is evidence evaluating the validity of similar tools based on direct observation in the clinical setting.^17^ Finally, the three scoring systems are all based on different scoring scales and comparison across scoring methods is limited.

## CONCLUSION

While this was a small pilot study, we found no significant correlation between SVI scores and neither faculty nor patient ratings of communication competencies. This raises concern about the validity of the SVI. Further, larger scale studies are needed to determine the best methods for assessing trainees’ communication skills and professionalism.

## Supplementary Information







## Figures and Tables

**Table 1:** Demographic characteristics and evaluation scores of emergency medicine (EM)-bound medical students rotating in an EM clerkship. Medians presented with interquartile range and minimum; means with standard deviation. None of the three scoring methods (faculty evaluation, CAT score, SVI score) had normal distributions (all p values <0.01).

Characteristics of Medical Students (n = 36)	Number
Male	20
Female	16
Medical schools represented	28
Evaluation Scores of Medical Students	Median, IQR (min)
Median number of faculty evaluations per student	9, 8–10 (6)
Median number of evaluations completed per attending	5, 2–8 (1)
Median faculty rating: professionalism	4, 4–5 (2)
Median faculty rating: patient care/communication	4, 4–5 (2)
Median CAT score	69, 66–70 (44)
Median SVI score	20,18–24 (14)

*IQR*, interquartile range; *CAT*, Communication Assessment Tool; *SVI*, Standardized Video Interview.
