# Supplementary material for: Standardized Video Interview Scores Correlate Poorly with Faculty and Patient Ratings
Source: West J Emerg Med. 2019 Dec 19;21(1):145–8. doi: 10.5811/westjem.2019.11.44054 (PMC6948708; doi:10.5811/westjem.2019.11.44054)
Supplement: Supplementary file 1 [file wjem-21-145-s001.docx]

Appendix A: Methodology for scoring the Standardized Video Interview.


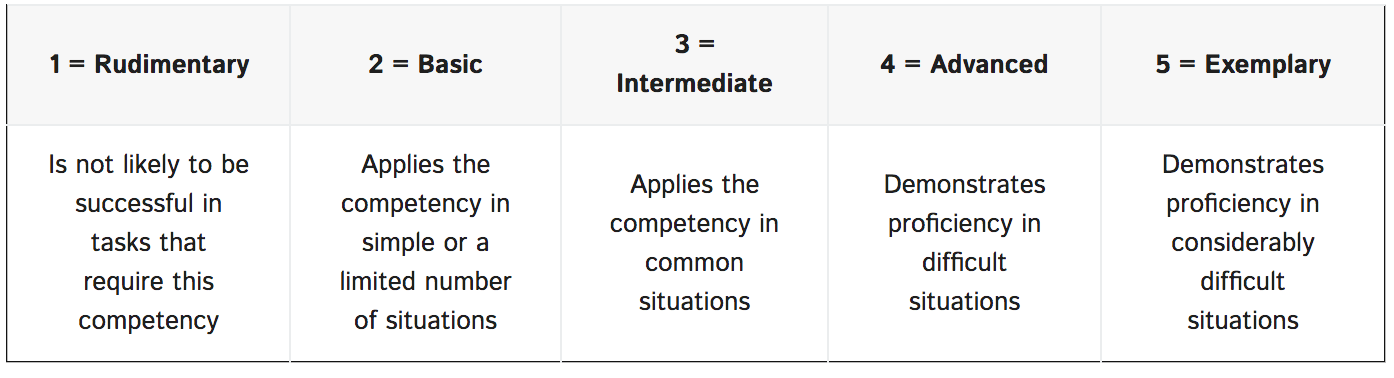


Total SVI score = Sum of the six individual question scores (minimum score 6, maximum 30).

Association of American Medical Colleges. How the SVI is scored. (<https://students-residents.aamc.org/applying-residency/article/how-svi-scored/>). Accessed February 26, 2018.
