# Supplementary material for: Standardized Video Interview Scores Correlate Poorly with Faculty and Patient Ratings
Source: West J Emerg Med. 2019 Dec 19;21(1):145–8. doi: 10.5811/westjem.2019.11.44054 (PMC6948708; doi:10.5811/westjem.2019.11.44054)

Appendix B: Screen shot of the scoring system at Beth Israel Deaconess Medical Center for faculty physician evaluation of medical student skill in the domains of professionalism and patient care/communication.


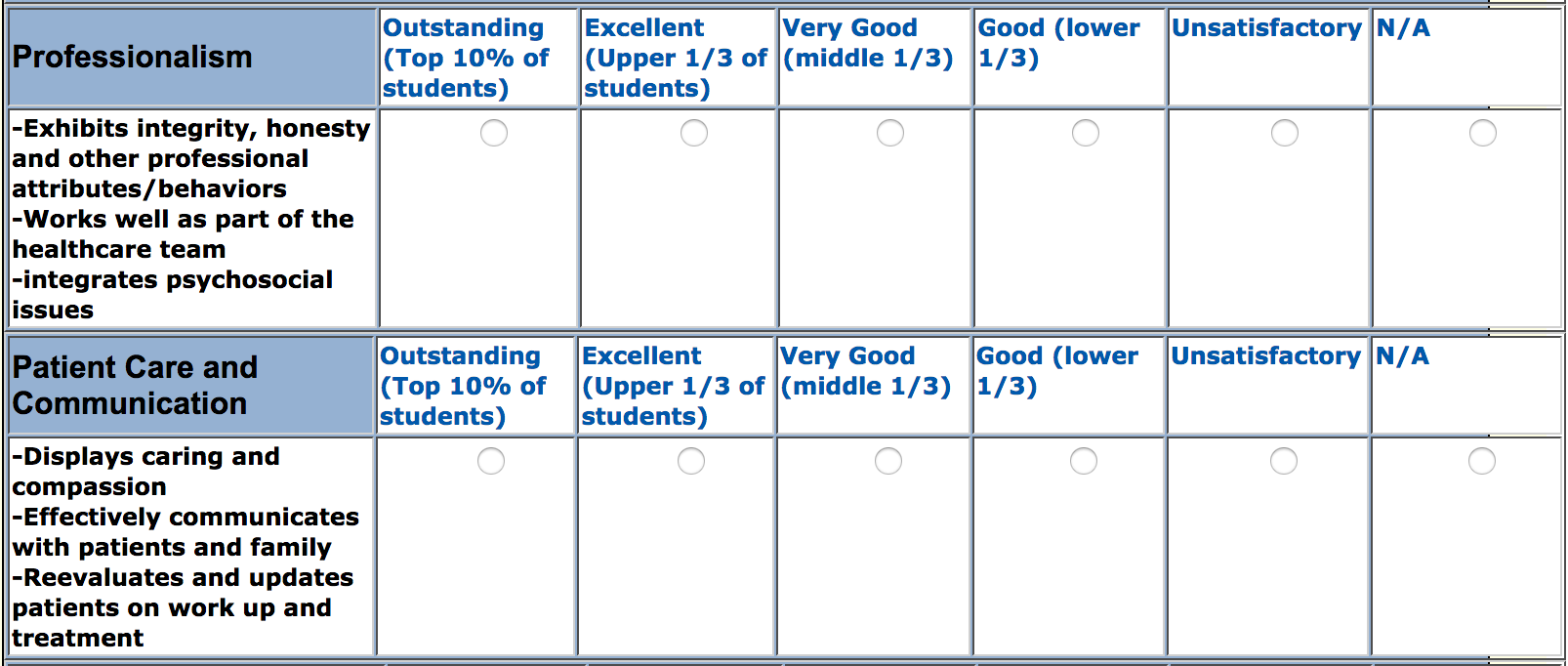

Supplement: Supplementary file 2 [file wjem-21-145-s002.docx]
