# Supplementary material for: Standardized Video Interview Scores Correlate Poorly with Faculty and Patient Ratings
Source: West J Emerg Med. 2019 Dec 19;21(1):145–8. doi: 10.5811/westjem.2019.11.44054 (PMC6948708; doi:10.5811/westjem.2019.11.44054)
Supplement: Supplementary file 3 [file wjem-21-145-s003.docx]

Appendix C: Scoring system for the Communication Assessment Tool.^1^


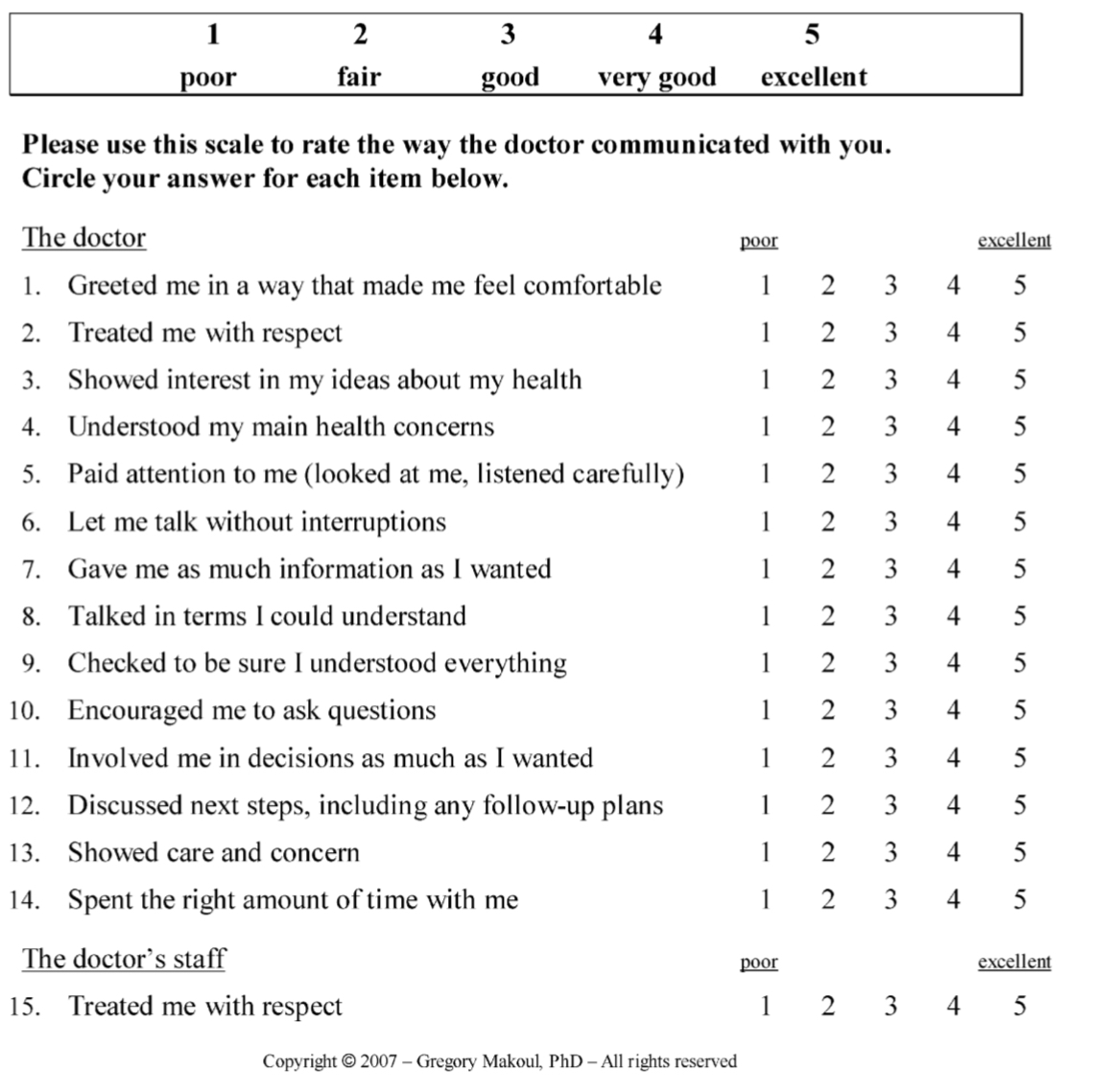


REFERENCE

^1^Makoul G, Krupat E, Chih-Hung C. Measuring patient views of physician communication skills: development and testing of the communication assessment tool. Patient Educ Couns. 2007;67:333-342.
